# Integrin αvβ3 Signaling in Tumor-Induced Bone Disease

**DOI:** 10.3390/cancers9070084

**Published:** 2017-07-08

**Authors:** Kristin A. Kwakwa, Julie A. Sterling

**Affiliations:** 1 Department of Veterans Affairs, Tennessee Valley Healthcare System, Nashville, TN 37212, USA; kristin.a.kwakwa@vanderbilt.edu; 2 Vanderbilt Center for Bone Biology, Vanderbilt University Medical Center, Nashville, TN 37232, USA; 3 Department of Cancer Biology, Vanderbilt University, Nashville, TN 37232, USA; 4 Department of Biomedical Engineering, Vanderbilt University, Nashville, TN 37235, USA; 5 Department of Medicine, Division of Clinical Pharmacology, Vanderbilt University Medical Center, Nashville, TN 37232, USA

**Keywords:** integrins, bone tumors, bone metastasis, tumor microenvironment

## Abstract

Tumor-induced bone disease is common among patients with advanced solid cancers, especially those with breast, prostate, and lung malignancies. The tendency of these cancers to metastasize to bone and induce bone destruction is, in part, due to alterations in integrin expression and signaling. Substantial evidence from preclinical studies shows that increased expression of integrin αvβ3 in tumor cells promotes the metastatic and bone-invasive phenotype. Integrin αvβ3 mediates cell adhesion to several extracellular matrix proteins in the bone microenvironment which is necessary for tumor cell colonization as well as the transmission of mechanical signals for tumor progression. This review will discuss the αvβ3 integrin receptor in the context of tumor-induced bone disease. Specifically, the focus will be the role of αvβ3 in modulating cancer metastasis to bone and tumor cell response to the bone microenvironment, including downstream signaling pathways that contribute to tumor-induced osteolysis. A better understanding of integrin dysregulation in cancer is critical to developing new therapeutics for the prevention and treatment of bone metastases.

## 1. Introduction

Advanced solid tumors frequently metastasize to bone, occurring in approximately 70–80% of patients with breast or prostate cancer, and in 30–40% of lung cancer patients [1]. Metastatic tumors disrupt normal bone remodeling to induce bone destruction by secreting factors (e.g., parathyroid hormone-related protein (PTHrP), interleukin-8, interleukin-11) that promote osteoclast formation. Subsequently, osteoclast-mediated bone resorption releases matrix-bound growth factors such as transforming growth factor beta (TGF-β), which further stimulate tumor growth and bone destruction [2,3]. Alternatively, metastatic tumors can secrete factors (e.g., bone morphogenetic proteins, insulin-like growth factors, endothelin-1) that promote osteoblast proliferation and differentiation, resulting in bone formation and sclerotic lesions [4]. This vicious cycle of tumor-induced bone disease (TIBD) results in severe comorbidities including extreme bone pain, spinal cord compression, hypercalcemia, and pathological fractures that significantly decrease patient quality of life and increase mortality [5,6,7]. Numerous preclinical studies have shown that the expression of specific integrin heterodimers, and their downstream signaling pathways, are perturbed in cancers that metastasize to bone. Most notably, integrin αvβ3 is upregulated in bone-metastatic tumor cells as well as multiple myeloma cells, and has been implicated in the progression of TIBD [8,9,10]. Interestingly, while integrin αvβ is also expressed in primary bone cancers such as osteosarcoma and chondrosarcoma, high αvβ3 expression has primarily been shown to promote metastasis of these tumors to the lung [11,12]. Hence, αvβ3 is a promising therapeutic target against bone metastases and the mechanisms by which it mediates the pathogenesis of secondary bone cancers and multiple myeloma are an area of extensive study [13]. This review will discuss integrin αvβ3 in the context of metastatic cancers in bone, particularly how αvβ3 modulates tumor cell response to the bone microenvironment as well as downstream signaling pathways that promote tumor-induced bone destruction.

## 2. The Biology of Integrin αvβ3

Integrin αvβ3 is a heterodimeric transmembrane glycoprotein that mediates cell adhesion to the extracellular matrix (ECM) through recognition of conserved arginine–glycine–aspartic acid (RGD) motifs in various ligands including osteopontin, vitronectin, and fibronectin [14]. Like other integrins, αvβ3 acts as a bidirectional signaling molecule. During “inside-out” signaling, adaptor proteins talin and kindlin bind the cytoplasmic tail of the β3 subunit, which not only links the integrin to the actin cytoskeleton but also causes conformational changes that increase its affinity for extracellular ligands [15,16]. In turn, ligation of activated αvβ3 triggers integrin clustering at the plasma membrane and recruitment of additional focal adhesion proteins (e.g., FAK, SFKs, paxillin, vinculin) which are important for actin cytoskeletal assembly as well as signal transduction (“outside-in” signaling) [17,18]. Integrin αvβ3 signaling is also modulated by lateral associations with growth factor receptors such as epidermal growth factor receptor (EGFR) [19] and TGF-β receptor II (TGFβRII) [20], and there is significant crosstalk between the downstream pathways (e.g., Ras-MEK-MAPK, PI3K-Akt, RhoA-ROCK) regulating cell migration, proliferation, and survival [21,22]. With respect to normal bone physiology, αvβ3 plays an important role in osteoclast-mediated bone resorption [23,24], angiogenesis [25,26], and phagocytosis of apoptotic cells [27].

## 3. Integrin αvβ3 Is Upregulated in Cancers that Metastasize to Bone

Metastasis is a multi-step process whereby cancer cells detach from the primary tumor, locally invade the surrounding tissue, transit through the vasculature or lymphatics, and colonize distant sites. Each stage of the metastatic cascade requires the activity of many different cell adhesion molecules, including integrins. Although several integrin heterodimers have been implicated in tumor cell interactions with the bone microenvironment (e.g., α2β1, α4β1, α5β1) [28], αvβ3 has been identified as a critical integrin for bone metastasis. Previous investigations have shown that the expression of integrin αvβ3 is increased in various bone-metastatic tumors such as breast, lung, and renal cancer compared to normal tissues [29]. One notable early study also demonstrated by immunohistochemistry that bone-residing metastases from breast cancer patients expressed higher levels of integrin αvβ3 compared to their respective primary tumors [30]. Collectively, these findings emphasize the importance of integrin αvβ3 in bone metastasis.

Another study illustrated that bone-metastatic subclones of a parental cancer cell line constitutively overexpressed integrin αvβ3 [31]. Specifically, a bone-tropic human breast cancer cell line (B02) was first established by repeated in vivo passages during which MDA-MB-231 breast carcinoma cells were injected into the left ventricle of the heart of nude mice and isolated from bone metastases [32]. The expression of various integrin heterodimers in these B02 cells was then assessed by immunoblotting and flow cytometry [31]. Results showed that integrin αvβ3 was overexpressed in B02 cells compared to the parental MDA-MB-231 cells while the cell surface expression of other integrins was not significantly different between the two cell lines.

In a more recent report, de novo expression of integrin αvβ3 in tumor cells that typically metastasize to the lungs was sufficient to promote homing to bone [33]. First, αvβ3 was exogenously expressed in the 66cl4 mouse mammary carcinoma cell line (66cl4beta3) and injected into the mammary fat pad of Balb/c mice. The 66cl4beta3-tumor bearing mice had significantly higher metastatic burden in the spine (20-fold increase) compared to mice that were inoculated with control 66cl4 cells. Spontaneous metastasis of 66cl4beta3 tumors to the long bones, particularly the femur, was also observed but these metastases were not detected in mice injected with control 66cl4 cells. Furthermore, several studies have shown that expression of functionally inactive αvβ3 mutants or treatment with αvβ3 antagonists significantly reduced the ability of tumor cells to colonize bone [9,31,34]. Taken together, these data demonstrate that integrin αvβ3 contributes to the osteotropism of metastatic cancer cells.

## 4. Expression of Tumor-Specific αvβ3 Promotes Bone Destruction

It is well-established that metastatic cancers induce osteoclastogenesis to initiate bone resorption, which facilitates tumor expansion in this metastatic niche [2,3,35]. Evidence from one preclinical study showed an increased number of osteoclasts adjacent to bone-residing tumors that overexpressed integrin αvβ3 [33]. In a previously described study, bone-metastatic human breast cancer cells that constitutively overexpressed αvβ3 (B02) induced significantly larger and more numerous osteolytic lesions in animals compared to the parental MDA-MB-231 cells from which they were derived [31]. In a later study by the same group, human MDA-MB-231 breast cancer cells were stably transfected to overexpress αvβ3 and subsequently injected into the tail vein of nude mice [36]. Mice bearing αvβ3-overexpressing tumors had significantly more bone destruction (2-fold increase) compared to mice inoculated with mock-transfected cells. Furthermore, treatment with the αvβ3 inhibitor PSK1404 significantly reduced the incidence of osteolysis in mice with αvβ3-overexpressing tumors. Interestingly, prostate cancer cells lacking integrin αvβ3 expression promote bone resorption while αvβ3-expressing prostate cancer cells stimulate bone formation, thus illustrating the role of αvβ3 in the development of osteoblastic lesions [9].

The molecular mechanisms by which tumor-specific αvβ3 promotes osteolysis are still being explored, but prior studies have shown that αvβ3 signaling resulted in the nuclear localization of transcription factors such as Runx2, which upregulated matrix metalloproteinases (e.g., MMP-9, MMP-13) and soluble receptor activator of NF-κB ligand (RANKL) to aid in bone matrix dissolution as well as osteoclast recruitment, differentiation, and function [37,38]. More importantly, integrin αvβ3 can augment TGF-β signaling [20] which has been shown to stimulate the expression of PTHrP by tumor cells and osteoblast expression of RANKL, thereby promoting osteoclast-mediated bone destruction [2,39]. In summary, these studies illustrate that increased αvβ3 expression in metastatic cancer cells contributes to the pathophysiology of tumor-induced bone destruction.

## 5. Integrin αvβ3 Modulates Tumor Response to the Rigid Bone Matrix

Over the past few decades, the ECM has been increasingly recognized as an important regulator of cell behavior and gene expression. For instance, matrix stiffness is increased in fibrotic soft tissues and has been linked to the malignant transformation of epithelial cells [40,41]. Matrix rigidity also stimulates integrin clustering, focal adhesion assembly, and RhoA-ROCK-dependent actomyosin contractility that can induce changes in gene expression. Mineralized bone is unique in that it has an elastic modulus ranging from 1.7 to 2.9 × 10^10^ Pa, which is orders of magnitude more rigid than soft tissues (10^2^–10^6^ Pa) [42,43]. One study explored the effects of bone matrix rigidity on metastatic tumors by culturing osteolytic MDA-MB-231 breast cancer cells and non-osteolytic MCF-7 cells on rigid bone-like substrates [44]. MDA-MB-231 cells significantly upregulated their expression of PTHrP (2.5-fold increase) and other genes involved in TIBD in response to substrate stiffness while MCF-7 cells showed no difference in PTHrP expression. Although tumor-specific integrins were not investigated, strong evidence indicated that the effects of substrate rigidity on PTHrP expression were mediated by mechanically transduced signals, particularly through activation of ROCK.

The mechanism by which matrix rigidity mediates osteolytic gene expression in metastatic tumors was further elucidated in a more recent study [45]. Specifically, metastatic breast (MDA-MB-231), prostate (PC-3), and lung (RWGT2) cancer cells cultured on bone-mimetic rigid substrates had increased expression of both integrin αvβ3 and PTHrP compared to cells cultured on more compliant substrates. Subsequently, fluorescence resonance energy transfer and co-immunoprecipitation assays were performed to investigate whether αvβ3, in addition to TGF-β, was regulating PTHrP expression. Results showed that colocalization of integrin αvβ3 and TGFβRII was significantly increased in tumor cells cultured on rigid substrates. The authors proceeded to demonstrate that rigidity-stimulated clustering of αvβ3 and TGFβRII activates Src which phosphorylates TGFβRII to induce p38 MAPK signaling and PTHrP expression. Inhibition of integrin αvβ3 in MDA-MB-231 cells using either an shRNA or the monoclonal antibody LM609 significantly decreased PTHrP expression. Furthermore, mice injected with MDA-MB-231 cells stably expressing shRNA against αvβ3 had reduced bone destruction. Collectively, these data indicate that crosstalk between integrin αvβ3 and TGFβ signaling modulates tumor cell response to the rigid bone microenvironment and promotes the transition of tumor cells to a bone-destructive phenotype.

## 6. Targeting Integrin αvβ3-Expressing Tumors in Bone

Currently, the standard of care for patients with TIBD are drugs that interfere with osteoclast-mediated bone resorption such as bisphosphonates [46] and RANKL inhibitors [47]. Clinical trials have demonstrated that these drugs are efficacious in reducing the frequency of skeletal-related events (SREs) (e.g., pathologic fractures, spinal cord compression, hypercalcemia) in patients with bone metastases [46]. However, there remains a need for therapies that directly target tumor cells residing in bone. Integrin αvβ3 is a promising therapeutic target for TIBD due to its high expression in metastatic tumors, angiogenic cells, and osteoclasts [48]; thus, αvβ3 antagonists could potentially disrupt multiple aspects of disease progression. Substantial evidence from preclinical investigations show that treatment with integrin αvβ3-targeting peptides (e.g., ATN-161, S247, cilengitide), non-peptide small molecules (e.g., PSK1404), or monoclonal antibodies (e.g., LM609) significantly reduces tumor growth and osteolysis in a variety of cancer types [34,36,45,49].

Several αvβ3-targeting drug candidates have advanced to clinical trials for the treatment of osteoporosis and cancer. The RGD-mimetic cyclic peptide cilengitide was first developed for treatment of glioblastoma multiforme [50,51] but has been investigated for use in patients with advanced solid tumors including prostate cancer, non-small cell lung cancer, and squamous cell carcinoma. The humanized monoclonal antibody etaracizumab was also in clinical trials for prostate cancer, ovarian cancer, and metastatic melanoma [52]. More recently, the small molecule GLPG0187 was evaluated for its effects in patients with progressive glioma and other advanced solid malignancies [53]. Despite success in early clinical trials, many of these therapies did not produce clinically relevant outcomes compared to standard chemoradiotherapy; however, few studies specifically targeted cancer patients with bone metastases. To evaluate the efficacy of novel or existing αvβ3 antagonists against bone metastases, future trials will need to be more inclusive of patients with TIBD.

## 7. Concluding Remarks

**Figure 1:**
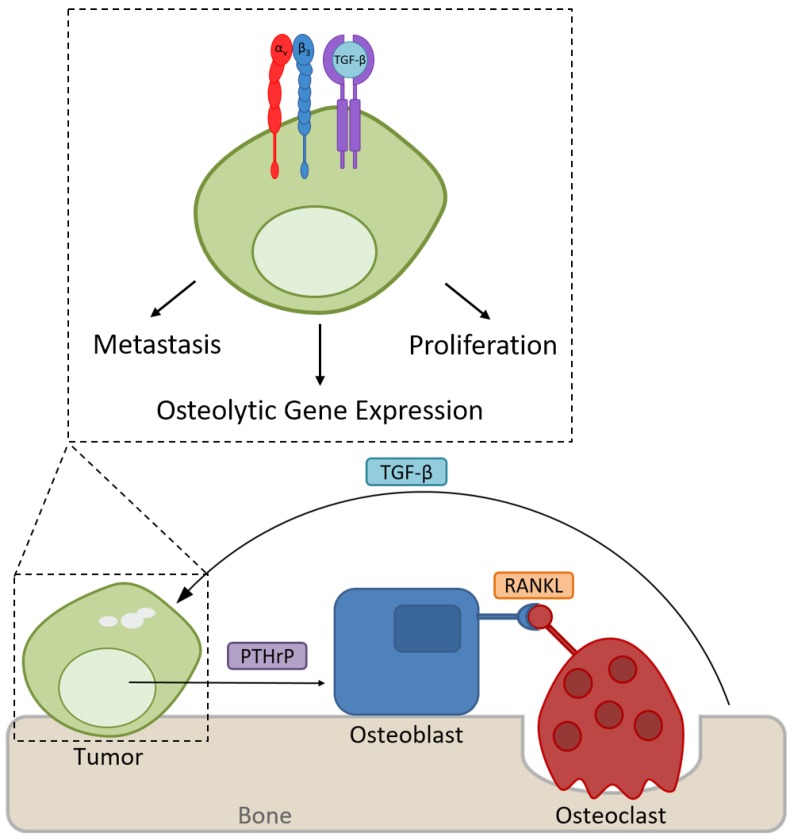
Expression of integrin αvβ3 promotes tumor growth and metastasis to bone. In the rigid bone microenvironment, αvβ3 interacts with TGFβRII to induce the expression of osteolytic genes such as PTHrP to stimulate osteoclast-mediated bone destruction.

Patients with advanced solid cancers frequently develop TIBD which involves growth of metastatic tumors in bone as well as osteoclast-mediated bone destruction. Despite palliative treatments, TIBD remains a highly debilitating disease for many cancer patients. Current therapies focus on inhibiting osteoclast-mediated bone resorption to reduce the risk of SREs, but there is a compelling need for therapies directly targeting metastatic tumor cells in bone. Despite the failure of existing drugs against advanced soft tissue tumors in clinical trials, integrin αvβ3 may be a promising therapeutic target for patients with TIBD as it is highly expressed in several bone-metastatic tumors including breast, prostate, and lung cancer. Preclinical studies have also demonstrated that the aberrant expression of tumor-specific αvβ3 promotes metastasis to bone, thereby increasing skeletal tumor burden and osteolysis. Mechanistically, integrin αvβ3 has been shown to mediate tumor cell response to the rigid bone microenvironment, which results in the upregulation of genes associated with bone destruction (Figure 1). Still, the exact mechanisms of integrin αvβ3 regulation in TIBD are not fully understood and the signaling pathways that are altered by changes in αvβ3 expression will need to be further explored in order to identify potential therapeutic targets. It is also important to note that because integrin αvβ3 is expressed by osteoclasts, proliferating endothelial cells, and certain immune cell populations, therapies that target αvβ3 may affect multiple aspects of TIBD in addition to bone resorption, including angiogenesis and inflammatory immune responses. Future studies will need to examine, in greater detail, the impact of integrin αvβ3 suppression on the tumor-bone microenvironment. A better understanding of integrin dysregulation in cancer and the mechanisms by which tumors respond to the bone microenvironment is crucial in order to develop novel therapeutics for the treatment of bone metastases.

## References

[1] Johnson R.W., Schipani E., Giaccia A.J. (2015). HIF targets in bone remodeling and metastatic disease. Pharmacol. Ther..

[2] Sterling J.A., Edwards J.R., Martin T.J., Mundy G.R. (2011). Advances in the biology of bone metastasis: How the skeleton affects tumor behavior. Bone.

[3] Buenrostro D., Mulcrone P.L., Owens P., Sterling J.A. (2016). The bone microenvironment: A fertile soil for tumor growth. Curr. Osteoporos. Rep..

[4] Logothetis C.J., Lin S.H. (2005). Osteoblasts in prostate cancer metastasis to bone. Nat. Rev. Cancer.

[5] Mundy G.R. (1997). Mechanisms of bone metastasis. Cancer.

[6] Roodman G.D. (2004). Mechanisms of bone metastasis. N. Engl. J. Med..

[7] Coleman R.E., McCloskey E.V. (2011). Bisphosphonates in oncology. Bone.

[8] Takayama S., Ishii S., Ikeda T., Masamura S., Doi M., Kitajima M. (2005). The relationship between bone metastasis from human breast cancer and integrin alphavbeta3 expression. Anticancer Res..

[9] McCabe N.P., De S., Vasanji A., Brainard J., Byzova T.V. (2007). Prostate cancer specific integrin alphavbeta3 modulates bone metastatic growth and tissue remodeling. Oncogene.

[10] Tucci M., De Palma R., Lombardi L., Rodolico G., Berrino L., Dammacco F., Silvestris F. (2009). Beta(3) integrin subunit mediates the bone-resorbing function exerted by cultured myeloma plasma cells. Cancer Res..

[11] Tome Y., Kimura H., Maehara H., Sugimoto N., Bouvet M., Tsuchiya H., Kanaya F., Hoffman R.M. (2013). High lung-metastatic variant of human osteosarcoma cells, selected by passage of lung metastasis in nude mice, is associated with increased expression of α(v)β(3) integrin. Anticancer Res..

[12] Lai T.H., Fong Y.C., Fu W.M., Yang R.S., Tang C.H. (2009). Stromal cell-derived factor-1 increase alphavbeta3 integrin expression and invasion in human chondrosarcoma cells. J. Cell. Physiol..

[13] Stucci S., Tucci M., Passarelli A., Silvestris F. (2015). Avβ3 integrin: Pathogenetic role in osteotropic tumors. Crit. Rev. Oncol. Hematol..

[14] Horton M. (1997). The αvβ3 integrin ‘vitronectin receptor’. Int. J. Biochem. Cell. Biol..

[15] Tadokoro S., Shattil S.J., Eto K., Tai V., Liddington R.C., de Pereda J.M., Ginsberg M.H., Calderwood D.A. (2003). Talin binding to integrin β tails: A final common step in integrin activation. Science.

[16] Ma Y.Q., Qin J., Wu C., Plow E.F. (2008). Kindlin-2 (Mig-2): A co-activator of beta3 integrins. J. Cell. Biol..

[17] Giancotti F.G., Ruoslahti E. (1999). Integrin signaling. Science.

[18] Qin J., Vinogradova O., Plow E.F. (2004). Integrin bidirectional signaling: A molecular view. PLoS Biol..

[19] Liu Z., Han L., Dong Y., Tan Y., Li Y., Zhao M., Xie H., Ju H., Wang H., Zhao Y. (2016). EGFRvIII/integrin β3 interaction in hypoxic and vitronectin enriching microenvironment promote GBM progression and metastasis. Oncotarget.

[20] Galliher A.J., Schiemann W.P. (2006). Beta3 integrin and Src facilitate transforming growth factor-beta mediated induction of epithelial-mesenchymal transition in mammary epithelial cells. Breast Cancer Res..

[21] Guo W., Giancotti F.G. (2004). Integrin signalling during tumour progression. Nat. Rev. Mol. Cell. Biol..

[22] Yeh Y.Y., Chiao C.C., Kuo W.Y., Hsiao Y.C., Chen Y.J., Wei Y.Y., Lai T.H., Fong Y.C., Tang C.H. (2008). TGF-beta1 increases motility and alphavbeta3 integrin up-regulation via PI3K, Akt and NF-kappaB-dependent pathway in human chondrosarcoma cells. Biochem. Pharmacol..

[23] Duong L.T., Lakkakorpi P., Nakamura I., Rodan G.A. (2000). Integrins and signaling in osteoclast function. Matrix Biol..

[24] McHugh K.P., Hodivala-Dilke K., Zheng M.H., Namba N., Lam J., Novack D., Feng X., Ross F.P., Hynes R.O., Teitelbaum S.L. (2000). Mice lacking beta3 integrins are osteosclerotic because of dysfunctional osteoclasts. J. Clin. Investig..

[25] Mahabeleshwar G.H., Feng W., Phillips D.R., Byzova T.V. (2006). Integrin signaling is critical for pathological angiogenesis. J. Exp. Med..

[26] Weis S.M., Cheresh D.A. (2011). AlphaV integrins in angiogenesis and cancer. Cold Spring Harb. Perspect. Med..

[27] Savill J., Dransfield I., Hogg N., Haslett C. (1990). Vitronectin receptor-mediated phagocytosis of cells undergoing apoptosis. Nature.

[28] Schneider J.G., Amend S.R., Weilbaecher K.N. (2011). Integrins and bone metastasis: Integrating tumor cell and stromal cell interactions. Bone.

[29] Vogetseder A., Thies S., Ingold B., Roth P., Weller M., Schraml P., Goodman S.L., Moch H. (2013). αv-Integrin isoform expression in primary human tumors and brain metastases. Int. J. Cancer.

[30] Liapis H., Flath A., Kitazawa S. (1996). Integrin αvβ3 expression by bone-residing breast cancer metastases. Diagn. Mol. Pathol..

[31] Pécheur I., Peyruchaud O., Serre C.M., Guglielmi J., Voland C., Bourre F., Margue C., Cohen-Solal M., Buffet A., Kieffer N. (2002). Integrin αvβ3 expression confers on tumor cells a greater propensity to metastasize to bone. FASEB J..

[32] Peyruchaud O., Winding B., Pécheur I., Serre C.M., Delmas P., Clézardin P. (2001). Early detection of bone metastases in a murine model using fluorescent human breast cancer cells: application to the use of the bisphosphonate zoledronic acid in the treatment of osteolytic lesions. J. Bone Miner. Res..

[33] Sloan E.K., Pouliot N., Stanley K.L., Chia J., Moseley J.M., Hards D.K., Anderson R.L. (2006). Tumor-specific expression of alphavbeta3 integrin promotes spontaneous metastasis of breast cancer to bone. Breast Cancer Res..

[34] Harms J.F., Welch D.R., Samant R.S., Shevde L.A., Miele M.E., Babu G.R., Goldberg S.F., Gilman V.R., Sosnowski D.M., Campo D.A. (2004). A small molecule antagonist of the αvβ3 integrin suppresses MDA-MB-435 skeletal metastasis. Clin. Exp. Metastasis..

[35] Thomas R.J., Guise T.A., Yin J.J., Elliott J., Horwood N.J., Martin T.J., Gillespie M.T. (1999). Breast cancer cells interact with osteoblasts to support osteoclast formation. Endocrinology.

[36] Zhao Y., Bachelier R., Treilleux I., Pujuguet P., Peyruchaud O., Baron R., Clement-Lacroix P., Clezardin P. (2007). Tumor alphavbeta3 integrin is a therapeutic target for breast cancer bone metastases. Cancer Res..

[37] Akech J., Wixted J.J., Bedard K., van der Deen M., Hussain S., Guise T.A., van Wijnen A.J., Stein J.L., Languino L.R., Altieri D.C. (2010). Runx2 association with progression of prostate cancer in patients: Mechanisms mediating bone osteolysis and osteoblastic metastatic lesions. Oncogene.

[38] Gupta A., Cao W., Chellaiah M.A. (2012). Integrin αvβ3 and CD44 pathways in metastatic prostate cancer cells support osteoclastogenesis via a Runx2/Smad 5/receptor activator of NF-κB ligand signaling axis. Mol. Cancer..

[39] Johnson R.W., Nguyen M.P., Padalecki S.S., Grubbs B.G., Merkel A.R., Oyajobi B.O., Matrisian L.M., Mundy G.R., Sterling J.A. (2011). TGF-beta promotion of Gli2-induced expression of parathyroid hormone-related protein, an important osteolytic factor in bone metastasis, is independent of canonical Hedgehog signaling. Cancer Res..

[40] Paszek M.J., Zahir N., Johnson K.R., Lakins J.N., Rozenberg G.I., Gefen A., Reinhart-King C.A., Margulies S.S., Dembo M., Boettiger D. (2005). Tensional homeostasis and the malignant phenotype. Cancer Cell..

[41] Levental K.R., Yu H., Kass L., Lakins J.N., Egeblad M., Erler J.T., Fong S.F., Csiszar K., Giaccia A., Weninger W. (2009). Matrix crosslinking forces tumor progression by enhancing integrin signaling. Cell.

[42] Guelcher S.A., Sterling J.A. (2011). Contribution of bone tissue modulus to breast cancer metastasis to bone. Cancer Microenviron..

[43] Sterling J.A., Guelcher S.A. (2011). Bone structural components regulating sites of tumor metastasis. Curr. Osteoporos. Rep..

[44] Ruppender N.S., Merkel A.R., Martin T.J., Mundy G.R., Sterling J.A., Guelcher S.A. (2010). Matrix rigidity induces osteolytic gene expression of metastatic breast cancer cells. PLoS One..

[45] Page J.M., Merkel A.R., Ruppender N.S., Guo R., Dadwal U.C., Cannonier S.A., Basu S., Guelcher S.A., Sterling J.A. (2015). Matrix rigidity regulates the transition of tumor cells to a bone-destructive phenotype through integrin beta3 and TGF-beta receptor type II. Biomaterials.

[46] Coleman R. (2011). The use of bisphosphonates in cancer treatment. Ann. N. Y. Acad. Sci..

[47] Fizazi K., Lipton A., Mariette X., Body J.J., Rahim Y., Gralow J.R., Gao G., Wu L., Sohn W., Jun S. (2009). Randomized phase II trial of denosumab in patients with bone metastases from prostate cancer, breast cancer, or other neoplasms after intravenous bisphosphonates. J. Clin. Oncol..

[48] Mulgrew K., Kinneer K., Yao X.T., Ward B.K., Damschroder M.M., Walsh B., Mao S.Y., Gao C., Kiener P.A., Coats S. (2006). Direct targeting of alphavbeta3 integrin on tumor cells with a monoclonal antibody, Abegrin. Mol. Cancer Ther..

[49] Khalili P., Arakelian A., Chen G., Plunkett M.L., Beck I., Parry G.C., Donate F., Shaw D.E., Mazar A.P., Rabbani S.A. (2006). A non-RGD-based integrin binding peptide (ATN-161) blocks breast cancer growth and metastasis in vivo. Mol. Cancer Ther..

[50] Reardon D.A., Fink K.L., Mikkelsen T., Cloughesy T.F., O'Neill A., Plotkin S., Glantz M., Ravin P., Raizer J.J., Rich K.M. (2008). Randomized phase II study of cilengitide, an integrin-targeting arginine-glycine-aspartic acid peptide, in recurrent glioblastoma multiforme. J. Clin. Oncol..

[51] Stupp R., Hegi M.E., Gorlia T., Erridge S.C., Perry J., Hong Y.K., Aldape K.D., Lhermitte B., Pietsch T., Grujicic D. (2014). Cilengitide combined with standard treatment for patients with newly diagnosed glioblastoma with methylated MGMT promoter (CENTRIC EORTC 26071–22072 study): A multicentre, randomised, open-label, phase 3 trial. Lancet Oncol..

[52] Hersey P., Sosman J., O'Day S., Richards J., Bedikian A., Gonzalez R., Sharfman W., Weber R., Logan T., Buzoianu M. (2010). A randomized phase 2 study of etaracizumab, a monoclonal antibody against integrin alpha(v)beta(3), + or −dacarbazine in patients with stage IV metastatic melanoma. Cancer.

[53] Cirkel G.A., Kerklaan B.M., Vanhoutte F., Van der Aa A., Lorenzon G., Namour F., Pujuguet P., Darquenne S., de Vos F.Y., Snijders T.J. (2016). A dose escalating phase I study of GLPG0187, a broad spectrum integrin receptor antagonist, in adult patients with progressive high-grade glioma and other advanced solid malignancies. Investig. New Drugs.

